# New Approaches Based on Inflammatory Indexes in the Evaluation of the Neoplastic Potential of Colon Polyps

**DOI:** 10.3390/life14101259

**Published:** 2024-10-02

**Authors:** Sedat Ciftel, Serpil Ciftel, Aleksandra Klisic, Filiz Mercantepe

**Affiliations:** 1 Department of Gastroenterology and Hepatology, Erzurum Training and Research Hospital, 25100 Erzurum, Turkey; sedat.ciftel@saglik.gov.tr; 2 Department of Endocrinology and Metabolism, Erzurum Training and Research Hospital, 25100 Erzurum, Turkey; serpil.ciftel@saglik.gov.tr; 3 Faculty of Medicine, University of Montenegro, 81101 Podgorica, Montenegro; aleksandranklisic@gmail.com; 4 Center for Laboratory Diagnostics, Primary Health Care Center, 81000 Podgorica, Montenegro; 5 Department of Endocrinology and Metabolism, Faculty of Medicine, Recep Tayyip Erdogan University, 53010 Rize, Turkey

**Keywords:** colorectal polyps, CALLY index, HALP score, immuno-inflammatory indexes

## Abstract

Colorectal polyps, precursors to colorectal cancer (CRC), require precise identification for appropriate diagnosis and therapy. This study aims to investigate the differences in hematological and inflammatory markers, specifically the CALLY index, HALP score, and immuno-inflammatory indexes, between neoplastic and nonneoplastic polyps. A retrospective cross-sectional study was conducted on 758 patients aged 61.0 ± 11.8 who underwent polypectomy between June 2021 and May 2024. Patients with colorectal adenocarcinoma (*n* = 22) were excluded. The polyps were classified into neoplastic and nonneoplastic categories based on histopathological evaluation. The study compared the CALLY index, HALP score, and various inflammatory indexes between neoplastic and nonneoplastic polyps. Out of 758 polyps analyzed, 514 were neoplastic, and 244 were nonneoplastic. Neoplastic polyps exhibited significantly lower CALLY and HALP scores (*p* < 0.05) and higher immuno-inflammatory indexes (*p* < 0.05) compared to nonneoplastic polyps. Dysplasia status, polyp diameter, and sigmoid colon localization were significant factors in determining neoplastic growth potential. No significant differences were observed in polyp localization in the proximal and distal colon segments or in solitary versus multiple polyps. The CALLY and HALP scores and immuno-inflammatory indexes can serve as valuable markers for distinguishing neoplastic from nonneoplastic polyps. These indexes reflect underlying inflammatory and immune responses, highlighting their potential utility in the early detection and risk stratification of colorectal polyps. Integrating these markers into clinical practice may enhance diagnostic accuracy and improve patient management, leading to timely interventions and better outcomes for individuals at risk of CRC.

## 1. Introduction

Colorectal polyps are often-occurring lesions in the gastrointestinal tract that can serve as precursors to colorectal cancer (CRC). The CRC is widely recognized as a significant contributor to global cancer mortality. Hence, precise identification and categorizing of colon polyps is crucial in defining appropriate diagnostic and therapeutic approaches. Inflammation, dietary conditions, immunological function, and heredity may all have an impact on the development of CRC [1]. Colon polyps are classified into two primary categories based on their histological characteristics: neoplastic and nonneoplastic. Neoplastic polyps can develop into cancerous growths and are referred to as adenomatous polyps. On the other hand, nonneoplastic polyps are typically categorized as inflammatory, hyperplastic, or hamartomatous polyps. It is crucial to distinguish between these two types of polyps to provide proper therapeutic care and to effectively manage patients through early endoscopic therapies [2]. It is now known that colorectal carcinomas can arise not only from conventional adenomas but also from serrated polyps and hyperplastic polyps [3].

Recent research has focused on examining the predictive significance of different hematological and inflammatory markers in cancerous conditions. In this particular situation, additional factors such as the platelet–lymphocyte ratio (PLR); CALLY index; hemoglobin, albumin, lymphocyte, platelet score (HALP); and immunological inflammatory indexes may serve as valuable aids in distinguishing between neoplastic and nonneoplastic polyps. The CALLY and HALP scores can indicate cell proliferation and differentiation in polyps and their nutrition status. Current studies support the critical role of inflammatory and thrombotic processes in all stages of colorectal cancer development. The systemic immune-inflammatory index (SIII) and PLR can provide insights into the inflammatory response and the extent of immune cell infiltration. The pan-immune inflammation value (PIV) is a newly identified biomarker that can potentially reflect the body’s immune response and systemic inflammatory status. It has been reported to have greater importance than the SIII in the prognosis of metastatic CRC [4].

The C-reactive protein (CRP) level in individuals with CRC is a clinical measure that indicates the extent of inflammation, according to reports [5]. The albumin level is a straightforward indicator of nutritional health. According to reports, low albumin and high CRP levels indicate inflammation and nutritional inadequacy [6]. The release of proinflammatory cytokines by the tumor can lead to chronic inflammation, which can disrupt the regeneration of lymphocytes by causing immunological dysregulation. Furthermore, tumor cells can exacerbate lymphocytopenia by attaching to lymphocyte receptors and triggering lymphocyte death through ligands. Additionally, it is believed that a reduction in the quantity of lymphocytes significantly influences the prognosis. A study examining systemic inflammatory markers for CRC risk discovered variations based on the cancer diagnosis and the timing of blood sampling. Elevated levels of the neutrophil–lymphocyte ratio (NLR), PLR, and SIII were associated with an increased risk, particularly in the year preceding cancer development [7]. The CALLY index, comprising CRP–albumin–lymphocyte parameters, was initially established by Hiroya lida and colleagues to indicate postoperative prognosis in patients with hepatocellular carcinoma [8]. The CALLY index, a prognostic indicator, has also been linked with overall survival in patients with CRC [9].

The HALP score has emerged as a novel prognostic biomarker in recent years, with applications in predicting clinical prognosis in different types of neoplasms. HALP is an innovative immuno-nutritional marker that integrates commonly measured indicators of the immunological state, such as platelet and lymphocyte counts, with nutritional status markers including albumin, and hemoglobin as an indicator of anemia. Initially, Chen et al. [10] pioneered the development of this method to forecast the prognosis of gastric cancer. In contrast to the CALLY index, the HALP score incorporates hemoglobin, which serves as a marker for anemia, into its computation. Anemia is a common occurrence in all cancer patients, particularly those with gastrointestinal malignancies. It has been reported that the HALP score may also be useful as a clinical prognostic factor in patients with CRC [11]. The PLR is becoming more popular as a biomarker for predicting cancer prognosis. This is due to the fact that patients with chronic inflammatory solid carcinoma often have enhanced platelet production. Additionally, platelets have a strong connection to the growth of cancer [12]. While the predictive significance of these indexes is established in individuals with colon cancer, their potential relevance in detecting malignancy before cancer diagnosis has not been adequately investigated. The objective of this study is to examine potential variations in indexes between neoplastic and nonneoplastic polyps during the initial phases of malignancy progression. Additionally, it is crucial to investigate whether these characteristics vary based on the presence and forms of dysplasia and the localization and quantity of polyps. These examinations are the initial stage in detecting neoplastic polyps and identifying high-risk patients. They also contribute to the prevention, treatment, and early intervention of colorectal cancer.

## 2. Participants and Methods

This retrospective cross-sectional study included patients undergoing colonoscopy from June 2021 to May 2024 who had at least one eligible colorectal polyp resected with polypectomy by an expert gastroenterologist. Colorectal adenocarcinoma was initially detected in 22 of 780 patients, and patients with colorectal cancer were excluded from the study. A total of 758 patients, comprising 303 women and 455 men with an average age of 61.0 ± 11.8 years, were included in our study.

The patients were instructed to keep on a watery diet for three days and to discontinue using aspirin or anticoagulants five days before the colonoscopy. All patients were prepared with 3–4 L of polyethylene glycol solution until clear rectal fluid was evacuated. The hot snare polypectomy method was used for pedunculated polyps, and saline solution mixed with epinephrine was pre-injected for nonpedunculated polyp resection. The colonoscopy procedures were performed with the Olympus-H180 AL (Tokyo, Japan) video colonoscope system with deep sedation monitored by an anesthesiologist. Snares were inserted through the channel of the colonoscope. Resected polyps were placed in a formalin container by an expert pathologist for histopathological examination. The localization, size, number, and pathology of the polyps were recorded. Blood samples were obtained simultaneously with colonoscopy. Biochemical and hematological parameters of the patients were recorded.

The indexes we used in our study were calculated with the following formulas [4,8,10]:

PLR = Platelet (10^9^/L)/Lymphocyte (10^9^/L)

CALLY Index = Albumin (g/L) × Lymphocyte (10^9^/L)/CRP (mg/dL) × 10^4^

HALP score = Hemoglobin (g/L) × Albumin (g/L) × Lymphocyte (10^9^/L)/Platelet(/L)

PIV = Neutrophil (10^9^/L) × Platelet (10^9^/L) × Monocyte (10^9^/L)/Lymphocyte (10^9^/L) (1)

SIII = Neutrophil (10^9^/L) × Platelet (10^9^/L)/Lymphocyte (10^9^/L)

SIRI = Neutrophil (10^9^/L) × Monocyte (10^9^/L)/Lymphocyte (10^9^/L)

We calculated the positive and negative predictive values (PPV, NPV) by using ROC analysis (Youden index calculation) with derived cut-off values for the new indexes.

The inclusion criteria were adult patients with colorectal polyps detected during colonoscopy who had undergone polypectomy and histopathological evaluation. The exclusion criteria were patients with cancer-detected polypectomy pathology, inflammatory bowel disease, polyposis syndromes, and rheumatological, hematological, infectious, liver, and malignant diseases that may have the potential to affect albumin and inflammatory parameters.

The study protocol received approval from the Ethics Committee of Erzurum Training and Research Hospital, with decision number 2022/15-153, dated 3 October 2022. The research was conducted in accordance with the ethical guidelines outlined in the Declaration of Helsinki. 

### Statistical Analysis

Statistical analyses were performed using IBM Statistical Package for Social Sciences software, version 22 (IBM Corp., Armonk, NY, USA). The Kolmogorov–Smirnov test and histograms were used to determine whether the variables were normally distributed or not. The Mann–Whitney U and Kruskal–Wallis tests were performed to compare with nonnormally distributed parameters. Student’s *t*-test was used for normally distributed parameters. Comparisons between categorical variables were performed with the Chi-square test. The nonnormally distributed data are represented by medians and quartiles. The normally distributed data are expressed as the mean ± standard deviation. Spearman Correlation analyses were performed to evaluate continuous variables. A statistically significant *p*-value was considered as *p* < 0.05. 

## 3. Results

Table 1 presents the results of the demographic analysis as well as the characteristics and localization of both neoplastic and nonneoplastic polyps. Out of the 780 polyps that were analyzed, 514 were found to be neoplastic, whereas 244 were nonneoplastic. The study excluded 22 patients whose pathology diagnosed colon adenocarcinoma. The prevalence of neoplastic polyps was 40.3% (207 individuals) among women and 59.7% (307 individuals) among men. The prevalence of nonneoplastic polyps was 39.3% (96 individuals) among women and 60.7% (148 individuals) among men. There was no statistically significant variation in the gender distribution across the groups (*p* = 0.807). Upon analyzing the dysplasia status of neoplastic polyps, it was found that 37.5% did not exhibit dysplasia, 49.0% had low-grade dysplasia, and 13.5% exhibited high-grade dysplasia. Nonneoplastic polyps showed no evidence of dysplasia in 95.1% of cases, whereas 4.5% exhibited low-grade dysplasia, and only 0.4% had high-grade dysplasia. There was a notable disparity between the groups regarding the occurrence of dysplasia (*p* < 0.001). A total of 81.1% of neoplastic polyps were pedunculated, whereas 18.9% were sessile. The percentages for nonneoplastic polyps were determined to be 75.0% and 25.0%, respectively. There was no statistically significant difference between the groups for the distribution of polyp pedunculation (*p* = 0.052). Among neoplastic polyps, 13.0% had diameters less than 5 mm, 40.3% between 5 and 10 mm, and 46.7% had diameters larger than 10 mm. In contrast, among nonneoplastic polyps, 21.7% had diameters less than 5 mm, 54.1% between 5 and 10 mm, and 24.2% had diameters greater than 10 mm. There was a notable disparity among the groups regarding the size of the polyp diameter (*p* < 0.001). Of all the neoplastic polyps, 20.8% were found in the proximal region, 67.1% in the distal region, and 12.1% in both the proximal and distal regions. On the other hand, 26.2% of the nonneoplastic polyps were located in the proximal region, 63.9% in the distal region, and 9.8% in both the proximal and distal regions. No statistically significant difference was observed between the groups regarding localization (*p* = 0.210). Rectum localization was observed in 26.5% of neoplastic polyps and 33.6% of nonneoplastic polyps. Furthermore, rectum localization was more prevalent in nonneoplastic polyps (n = 0.042). This study indicated that sigmoid localization was observed in 45.1% of neoplastic polyps and 33.6% of nonneoplastic polyps. Additionally, sigmoid localization was more frequently observed in neoplastic polyps compared to nonneoplastic polyps (*p* = 0.003). The locations of the descending colon, splenic flexure, transverse colon, hepatic flexure, ascending colon, and cecum did not show any significant differences between neoplastic and nonneoplastic colon polyps (*p* ≥ 0.05). These data suggest that characteristics such as dysplasia status, polyp diameter, and sigmoid colon placement have a significant role in determining the likelihood of developing neoplastic growth.

Table 2 shows the allocation of cancerous and noncancerous growths. The distributions indicate that tubular adenomas are the predominant type of neoplastic polyps, whereas hyperplastic polyps are the most frequent nonneoplastic polyps. Furthermore, neoplastic polyps were also discovered to include less prevalent kinds, such as villous and serrated adenomas. A significant proportion of nonneoplastic polyps consisted of inflammatory polyps. Table 3 compares neoplastic and nonneoplastic polyps regarding age, diabetes mellitus, metabolic parameters, and lymphocyte indexes. The average age in the neoplastic polyp group was 61.6 ± 11.2 years, while the average age in the nonneoplastic polyp group was 59.8 ± 12.9 years. There was no significant difference in age between the two groups (*p* = 0.070). There was no difference in the frequency of diabetes between the neoplastic and nonneoplastic polyp groups (24.1% and 23.8%, respectively). No statistically significant differences were seen between the groups regarding glucose, total protein, neutrophil count, and platelet count (*p* = 0.691, *p* = 0.170, *p* = 0.368, and *p* = 0.876, respectively). Statistically significant differences were seen between the neoplastic and nonneoplastic polyp groups in terms of albumin, CRP, lymphocyte, PLR, CALLY index, HALP score, PIV, SIII, and SIRI (*p* < 0.001, *p* < 0.001, *p* < 0.001, *p* < 0.001, *p* < 0.001, *p* = 0.013, *p* = 0.003, and *p* = 0.007, respectively).

The polyps were categorized into three groups based on the presence and severity of dysplasia: those without dysplasia, those with low-grade dysplasia, and those with high-grade dysplasia. These groups were then compared in terms of several clinical and biochemical markers. The findings of this comparison are displayed in Table 4. The mean ages did not differ significantly among the groups (*p* = 0.182), with mean ages of 60.8, 60.7, and 63.7 years, respectively. The levels of glucose, total protein, CRP, neutrophils, and platelets were comparable among all groups, and no statistically significant differences were detected (*p* = 0.510, *p* = 0.343, *p* = 0.116, *p* = 0.311, and *p* = 0.670, respectively). Nevertheless, there were notable variations between the three groups with respect to albumin, lymphocytes, PLR, CALLY index, HALP score, PIV, SIII, and SIRI (*p* < 0.001, *p* < 0.001, *p* < 0.001, *p* = 0.003, *p* < 0.001, *p* = 0.017, *p* < 0.001, and *p* < 0.001, respectively).

Table 5 shows the comparison of the PLR, CALLY index, HALP score, PIV, SIII, and SIRI parameters based on the location of polyps in the proximal colon, distal colon, and both colon segments. None of these analyzed indicators exhibited a noteworthy disparity across all three segments of the colon. Similarly, the data in Table 6 showed no significant differences in the PLR, CALLY index, HALP score, PIV, SIII, and SIRI parameters when comparing polyps based on whether they were solitary or multiple.

To determine that polyp size is related to the inflammatory indexes utilized in our investigation, we established a cut-off value of 1 cm for polyp size. We compared the PLR, CALLY index, HALP score, PIV, SIII, and SIRI between polyps measuring ≥1 cm and those measuring <1 cm. The findings are presented in Table 7. The HALP score was considerably lower in colon polyps measuring ≥1 cm (*p* = 0.036). The CALLY index decreased in this group; however, no statistically significant difference was seen. The PIV, SIII, and SIRI levels increased in the ≥1 cm group, but no statistically significant difference was seen.

Logistic regression analysis evaluated the risk factors for developing neoplastic polyps (Table 8). In this study, bigger polyp diameter (*p* < 0.001, OR = 2.721, 95% CI 1.624–4.559), lower CALLY index (*p* = 0.008, OR = 0.927, 95% CI 0.876–0.980), and lower HALP score (*p* = 0.035, OR = 0.992, CI 0.986–0.999) all pointed to the growth of a neoplastic polyp. The PIV, SIII, and SIRI did not affect neoplastic polyp development (*p* = 0.269, *p* = 0.413, and *p* = 0.870, respectively). For every index analyzed in this current study, we calculated the PPV and NPV values (Table 9). Although the PPV for all indexes was above 70%, a small NPV was detected for all parameters (below 40%). 

## 4. Discussion

In the present study, the potential of using various indexes reflecting inflammation and immune status to assess colon polyps’ neoplastic potential was investigated in addition to demographic and biochemical parameters. Significant differences were found between neoplastic and nonneoplastic polyps regarding hematological and biochemical parameters and lymphocyte indexes. In particular, inflammatory parameters such as CRP, albumin, PLR, CALLY index, HALP score, PIV, SIII, and SIRI were significantly different in neoplastic polyps compared to nonneoplastic polyps.

In the present study, both neoplastic and nonneoplastic polyps were found at similar rates in men and women, indicating that gender is not a significant risk factor in polyp characteristics. However, it was found that the frequency of polyps was generally higher in men. This finding, in line with data reported in previous studies, supports the effect of gender on the incidence of colon polyps while also highlighting the limited effect of gender on polyp characteristics. Colon polyps can be considered as precursor lesions of colon cancer. Gilbertson first suggested in the 1960s that colorectal cancer may arise from intermediate lesions in the colon [13]. In the late 1980s, Fearon and Vogelstein [14] described CRC as a genetic disease in which the progression from polyp to carcinoma is a sequence of specific genetic mutations. 

The clinical importance of polyps is due to the fact that more than 95% of colon adenocarcinomas arise from polyps [15]. The vast majority of colorectal cancers develop from precancerous adenomatous or serrated polyps. It is the third most commonly diagnosed cancer in men in the United States. It is also the most common type of cancer diagnosed in men, with the most recent data in the United States showing an incidence rate of colorectal cancer in men of 42 cases per 100,000 men per year. This rate is lower in women, at 32 cases per 100,000 women. Beginning at age 50, the incidence is approximately 30% higher in men than women [16].

The prevalence of colon adenomas is higher in men. In a cross-sectional study, the risk of adenomas in screening colonoscopy was found to be 1.77 times higher in men than in women [17]. The same study reported that colon adenomas increased significantly in both genders with increasing age. In a multicenter study conducted in Turkey with 6508 patients, the frequency of colon polyps was found to be 37% in male patients and 27% in female patients. The frequency of CRC was found to be 3.8% in males and 1% in females [18]. Therefore, it is an expected finding that colon polyps are more frequently observed in men. Based on this information, we can conclude that neoplastic polyps, which have a higher malignant potential, should be more frequently seen in men. However, although both neoplastic and nonneoplastic polyps are more frequently seen in men when both sexes are evaluated within themselves, no gender-specific difference was found between them. 

The findings of this study show that neoplastic polyps exhibit a greater proportion of high-grade dysplasia than nonneoplastic polyps. This is an expected finding. Most neoplastic polyps are adenomatous polyps. The significance of these polyps is that they undergo malignant degeneration. The size, histologic type, and degree of dysplasia of adenomas determine their malignant potential. Histologically, conventional adenomas are divided into tubular, tubulovillous, or villous histologic subtypes. As the degree of dysplasia and polyp size increase, the potential for malignant transformation of polyps also increases. Polyps smaller than 10 mm rarely and very slowly can become cancerous. Adenomas larger than 10 mm, villous in structure, or with high-grade dysplasia are at the highest risk of malignant transformation. A total of 1.7% of adenomas measuring 1–5 mm, 6.6% of adenomas measuring 6–9 mm, and 30.6% of adenomas measuring larger than 10 mm have advanced histologic features and have the potential to become cancerous [19]. Although nonneoplastic polyps generally have a low risk of becoming cancerous, this risk may increase under certain genetic conditions or in some exceptional cases. Therefore, correct identification of polyps and regular follow-up is essential. Hyperplastic polyps are the most common nonneoplastic polyps. The absence of atypia in the cells is an important point; therefore, they are considered not neoplastic. This is the point that is under debate. Today, it is reported that hyperplastic polyps carry a risk of cancer or indicate an increased risk of cancer in the presence of certain conditions [20]. Genetic studies have also shown that specific genetic changes (e.g., microsatellite instability—MSI) can be seen in hyperplastic polyps that are located in the right colon and reach diameters greater than 10 mm and that the risk of cancer development from such hyperplastic polyps will increase [21]. The results of our study show that neoplastic polyps have higher dysplasia degrees and larger sizes. In addition, no difference was found between neoplastic and nonneoplastic polyps regarding polyp stem and whether the polyps are located in the proximal or distal colon. However, nonneoplastic polyps are seen to be located in the rectum, while neoplastic polyps are more frequently located in the sigmoid region. In a study on the localization of colon polyps, generally, all types of polyps were found to be more frequent in the distal colon [22]. In another study, it was found that the most common location for polyps was the sigmoid colon region, but this study also showed that the pathology of the polyp was not related to the colon region [23]. In a study on determining high-malignant risk colorectal polyps, it was reported that the malignant potential was higher in the left colon (especially sigmoid and rectum) than in the right colon and that the risk of malignancy increased due to the risk of histological invasion when the polyp was sessile, in line with other studies [24]. In our study, neoplastic polyps were mainly located in the sigmoid colon.

Polyps are usually asymptomatic and are usually discovered incidentally during screening colonoscopies for CRC. However, patients may present with bright or dark red painless rectal bleeding, effacement, or mixed or dripping stools. Other presentations include diarrhea, constipation, abdominal pain, mucus in the stool, or symptoms and signs of iron deficiency anemia due to chronic bleeding [15]. In our study, hemoglobin and ferritin values were found to be similar among polyp types. In addition, these parameters were not associated with the degree of dysplasia. This finding demonstrates that although it is known that polyps can present with bleeding, their malignant potential is unrelated to anemia parameters. 

In our study, it was found that glucose levels and DM incidence were not associated with the type of polyp and the presence of dysplasia. Literature studies have found that the risk of colorectal polyps in patients with type 2 DM increased by 1.23 times and that the incidence of colon polyps increased with the duration of diabetes [25]. However, to our knowledge, there is no study in the literature investigating the presence of DM in terms of polyp types.

However, a recent meta-analysis showed that patients with T2DM have a 30% higher risk of CRC than the general population [26]. Other studies have also shown that the risk of CRC is high in diabetic patients [27]. The difference may not have been found because our research was conducted in a single center, and both risk and protective factors such as smoking, alcohol, diet, medications, and NSAIDs (non-steroidal anti-inflammatory drugs) were not included in this study.

In this study, the use of inflammatory indexes in the evaluation of neoplastic and dysplastic potential of colon polyps was investigated. Our findings are consistent with the data in the current literature and support the role of inflammation in neoplastic processes. The fact that CRP, an important indicator of systemic inflammation, is found to be higher in neoplastic polyps suggests that they may be related to the inflammatory response. In addition, the low albumin levels in individuals with neoplastic polyps indicate that this parameter can also be used as a marker. Especially in the high-grade dysplasia group, the low albumin level may indicate an increase in inflammatory processes and protein catabolism. Indexes such as the PLR, CALLY index, HALP score, PIV, SIII, and SIRI are accepted as indicators of both inflammation and immune response. The fact that these indexes are higher in neoplastic polyps suggests that these polyps may be associated with inflammatory and immune responses. In particular, indexes such as the HALP score and CALLY index stand out as new parameters that may be important in evaluating neoplastic potential. 

Many of the inflammation indexes we investigated were constructed on a lymphocyte basis. Lymphopenia can occur due to primary conditions such as congenital immunodeficiency disorders or acquired causes such as malnutrition, infectious diseases, sepsis, autoimmune and lymphoproliferative disorders, malignancies, drugs (steroids, chemotherapy), and protein-losing conditions such as severe burns, amyloidosis, and inflammatory bowel disease. Increasing evidence suggests that cancer progression is influenced by the systemic inflammatory response [28]. Immune modulators secreted by tumor cells, including TGF-β, IL-10, and CRP, impair lymphocyte action in systemic inflammation [29]. These cytokines and immunosuppressive factors released by the tumor can reduce the activity and number of lymphocytes. Chronic inflammation caused by malignancy can cause lymphocytes to be depleted and reduced by apoptotic processes. Tumor-infiltrating lymphocytes, such as natural killer and T helper type 1, are effective components against cancer growth and metastasis in various cancers through the production of interferon-gamma. The lymphocytic response is a key component of controlling cancer progression. Increased lymphocyte infiltration is considered an independent pretreatment neoadjuvant therapy response parameter in breast cancer [30]. CD8(+) T cells constitute the major effective cell group against cancer cells. To date, the prognostic importance of the presence of CD8(+) T lymphocytes in colorectal cancer has been demonstrated in many studies, with better survival outcomes demonstrated by more CD8(+) tumor-infiltrating lymphocytes [31]. The initial immune response to an early neoplasm is thought to reflect the response to acute tissue injury, with sequential infiltration by various myeloid populations leading to eventual infiltration by lymphocytes. Inflammation and the accompanying increase in stress may lead to increased cortisol levels, which may contribute to decreased lymphocyte numbers and function [32].

The relationship between inflammation and the development of neoplastic diseases continues to be the subject of many studies. The first evidence for this emerged in 1828 when the French surgeon Jean Nicholas Marjolin observed the development of squamous cell carcinoma around an open wound [33]. One of the closest relationships between chronic inflammation and cancer is observed in inflammatory bowel disease, where a 10-unit increase in the endoscopic inflammation score doubles the risk of colorectal cancer [33]. In the study conducted by Ruiya et al. [34], the preoperative CALLY index decreased significantly as the esophageal Ca stage increased. Moreover, patients with a decreased CALLY index had lower overall survival. A study conducted by Takeda et al. [35] on colorectal cancer reported that the CALLY index was an independent prognostic biomarker in these patients. In fact, in a study conducted by Furukawa et al. [9] on patients with colorectal cancer with liver metastases, there were more postoperative complications after metastasectomy in those with a low CALLY index. In a study conducted by Feng et al. [36], some parameters related to inflammation were reported as potential indicators that could help in the diagnosis of colorectal cancer. In their study, the early colorectal cancer group and the adenomatous polyp group were compared in terms of inflammatory parameters; the PLR and SIII were higher, hemoglobin and albumin were lower in the early colorectal cancer patients, and the average age of cancer patients was older than the other group, unlike in our study.

Colorectal cancers are more common in the elderly population. Approximately 80% of all new cases are diagnosed in individuals aged 55 years and older, and the median age at diagnosis in industrialized countries such as the United States is approximately 70 years. In our study, the age groups were similar regarding polyp type and dysplasia. This was probably because our patients did not have colorectal cancer. The transformation from adenomas to cancer occurs over a relatively long period. 

The size of a polyp is regarded as a significant determinant in assessing the malignant potential of non-sessile polyps [2,37]. The increased prevalence of neoplastic polyps measuring ≥1 cm in our study results aligns with this information. Still, the fact that there was no link between polyp size and other indexes (PLR, CALLY index, PIV, SIII, and SIRI) shows that neoplastic potential is linked to the inflammatory indexes used in this study, no matter how big the polyp is. The relationship between the HALP score and polyp size was statistically very low in significance.

A study [38] reported an inflammatory polyp frequency of 2.9% among all polyps and 12% among nonneoplastic polyps, but our investigation revealed a frequency of 9.3% in all polyps and 29% in nonneoplastic polyps. Numerous variables contribute to the genesis of inflammatory polyps. In addition to inflammatory bowel disease, it may arise from chronic inflammation or tissue injury, environmental influences, dietary practices, tobacco and alcohol consumption, persistent intestinal infections, obesity, and the aging process. While we removed inflammatory bowel disease and other inflammatory rheumatological conditions from our investigation, additional factors were not excluded. In this community, encompassing the region where our study was performed, intestinal infections are prevalent due to smoking, obesity, and substandard living conditions, with a mean patient age of 61.0 ± 11.8 years. The explanations mentioned above may account for the elevated incidence of hyperplastic polyps observed. Moreover, our hospital is equipped with state-of-the-art facilities that provide a comprehensive view of the region, and the pathology reports at our center have been systematically reviewed by two pathologists. Nonetheless, no prior research exists in the literature with which we may compare the prevalence of polyps in our location. In this regard, our research is the inaugural investigation on this topic in our region.

The results of our study show that inflammatory indexes are useful for evaluating colon polyps’ cancerous and precancerous potential. We posit that these indicators possess the potential to furnish valuable insights for the prompt identification, monitoring, and treatment of neoplastic and dysplastic polyps in clinical settings. It is important to consider the positive and negative aspects of our study while assessing its quality. The current study analyzed a total of 758 patients with polyps. This substantial sample size allowed for improved generalization of the results to the overall population and enhanced the reliability of the statistical analyses. The study assessed many inflammatory markers, including CRP, PLR, CALLY index, HALP score, PIV, SIII, and SIRI. This condition elucidated the correlation between inflammation and immune response in neoplastic polyps more thoroughly. An in-depth analysis of neoplastic and nonneoplastic polyps identified distinct biochemical and inflammatory disparities between these two categories. This led to the discovery of novel biomarkers for assessing the cancerous potential of polyps. Our study assessed the clinical use of novel indexes, namely, the HALP score and CALLY index, which have not been extensively investigated in the existing literature on colon polyps. An analysis of these indexes may offer novel strategies for clinical practice. The obtained data are derived from standard biochemical and hematological indicators and have the potential to provide therapeutically meaningful information for the early detection and treatment of neoplastic polyps. A significant advantage of investigating these inflammatory indexes in colon polyps is that they are simple, inexpensive, and accessible instruments. They can be used to determine the malignant potential of polyps and are easily integrated into clinical procedures. Furthermore, our study assessed inflammatory indicators and biochemical and demographic factors, including age and gender. This allowed us to conduct a comprehensive assessment of the cancerous potential of polyps. Although our study has numerous merits, it also possesses drawbacks. Our work is designed retrospectively, which introduces potential biases. For instance, certain inflammatory and immunological states could have influenced the relationship between the inflammatory indexes and neoplastic polyps, causing confounding effects. Nevertheless, as the anesthesiologist thoroughly assessed the patients who underwent colonoscopy before the operation, we believe that these factors did not substantially impact the study findings. This study was conducted in a single center, ensuring homogeneity in the study population. However, this may restrict the generalizability of the findings to other populations. A significant limitation of our study is the inability to assess the long-term impact of these indexes on predicting the probability of polyp malignant transformation, as there was a lack of long-term polyp follow-up. Furthermore, the precise threshold values of the inflammatory indexes employed in the study to predict the likelihood of malignancy have not been established. To our knowledge, the indexes examined in this study regarding colon polyps have not been previously addressed in the literature. This has hindered the complete comparison of our results with other similar research in the existing literature.

## 5. Conclusions

The use of the CALLY index, HALP score, and immuno-inflammatory indexes in clinical practice can contribute to risk stratification of colorectal polyps, potentially reducing the incidence of colorectal cancer (CRC) through timely interventions. When polyps are detected in patients undergoing endoscopic procedures, examining the inflammation indexes may be important in terms of providing an idea to the endoscopist about what kind of polypectomy method will be used for the polyp during the procedure and in planning the treatment and follow-up and in terms of giving a preliminary idea to the pathologists who will evaluate the polypectomy material in the process of differentiating the polyp from neoplastic or nonneoplastic. These markers improve diagnostic accuracy and offer a cost-effective method for identifying high-risk patients, promoting public health initiatives aimed at CRC prevention. This study also presents the impact of dysplasia, aging, and colon polyp localization on many measures of inflammatory and immunological responses. The involvement of inflammatory and immunological responses seems crucial in the progression of dysplasia and aging. Our study’s results can offer significant insights for future investigations aiming to enhance comprehension of the impact of inflammation in clinical settings and for devising approaches for the prevention and treatment of disorders such as dysplasia.

## Figures and Tables

**Table 1:** Comparison of neoplastic and nonneoplastic polyp groups in terms of demographic findings, polyp characteristics, and localization.

	Neoplastic Polyps (n = 514)	Nonneoplastic Polyps (n = 244)	Total (n = 758)	*p*
**Gender**				0.807
Female	207 (40.3)	96 (39.3)	303 (40.0)	
Male	307 (59.7)	148 (60.7)	455 (60.0)	
**Dysplasia**				0.001 *
No dysplasia	193 (37.5)	232 (95.1)	425 (56.1)	
Low-Grade Dysplasia	252 (49.0)	11 (4.5)	263 (34.7)	
High-Grade Dysplasia	69 (13.5)	1 (0.4)	70 (9.2)	
**Polyp stalk**				0.052
Pedunculated polyps	417 (81.1)	183 (75.0)	600 (79.2)	
Sessile polyps	97 (18.9)	61 (25.0)	158 (20.8)	
**Polyp Diameter**				0.001 *
<5 mm	67 (13.0)	53 (21.7)	120 (15.8)	
5–10 mm	207 (40.3)	132 (54.1)	339 (44.7)	
≥10 mm	240 (46.7)	59 (24.2)	299 (39.5)	
**Localization**				0.210
Proximal Colon	107 (20.8)	64 (26.2)	171 (22.6)	
Distal Colon	345 (67.1)	156 (63.9)	501 (66.1)	
Proximal + Distal Colon	62 (12.1)	24 (9.8)	86 (11.3)	
Rectum	136 (26.5)	82 (33.6)	218 (28.8)	0.042 *
Sigmoid colon	232 (45.1)	82 (33.6)	314 (41.4)	0.003 *
Descending colon	69 (13.4)	33 (13.5)	102 (13.5)	0.970
Splenic flexure	39 (7.6)	15 (6.1)	54 (7.1)	0.471
Transvers colon	70 (13.6)	36 (14.8)	106 (14.0)	0.674
Hepatic flexure	31 (6.0)	9 (3.7)	40 (5.3)	0.178
Ascending colon	39 (7.6)	20 (8.2)	59 (7.8)	0.770
Cecum	33 (6.4)	17 (7.0)	50 (6.6)	0.777

* *p* < 0.05 is statically significant (bold).

**Table 2:** Distribution of neoplastic and nonneoplastic polyps.

Neoplastic Polyps	n (%)	Nonneoplastic Polyps	n (%)
Tubular adenoma	378 (73.6)	Hyperplastic polyps	165 (67.6)
Tubulovillous adenoma	116 (22.5)	Inflammatory polyps	71 (29.0)
Villous adenoma	18 (3.5)	Others (Ksantamatose, lymphoid, hamartomatous)	8 (3.4)
Serrated adenoma	2 (0.4)		
**Total**	**514**		**244**

**Table 3:** Comparison of neoplastic and nonneoplastic polyp groups in terms of age, diabetes mellitus, biochemical parameters, and lymphocyte-based indexes.

	Neoplastic Polyps (n = 514)	Nonneoplastic Polyps (n = 244)	Total (n = 758)	*p*
**Age (year)**	61.6 ± 11.2	59.8 ± 12.9	61.0 ± 11.8	0.070
**Glucose (mg/dL) (mean ± SD)**	107 (±45)	108 (±44)	107 (±45)	0.691
**DM n (%)**	124 (24.1)	58 (23.8)	182 (24)	0.915
**Total protein (g/L)**	68 (36–84)	69 (46–89)	68 (36–89)	0.170
**Albumin (g/L)**	42 (19–52)	44 (26–53)	42 (19–53)	0.001 *
**CRP (mg/dl)**	5.9 (0.1–161)	3.2 (0.0–138)	5 (0–161)	0.001 *
**Hemoglobin (g/dL), median (min-max)**	14.2 (4.6–19.7)	14 (6.2–18.5)	14.1 (4.6–19.7)	0.989
**Ferritin (ng/mL), median (min-max)**	51 (1–1501)	54.4 (1.9–1650)	52 (1–1650)	0.823
**Neutrophil (10^9^/L)**	4.46 (1.58–28)	4.32 (0.66–14.4)	4.42 (0.66–28.3)	0.368
**Lymphocyte (10^9^/L)**	2.14 (0.19–6.80)	2.37 (0.49–6.68)	2.20 (0.19–6.80)	0.001 *
**Platelet (10^9^/L)**	271 (92–677)	270 (91–759)	270 (91–759)	0.876
**PLR**	128 (24–810)	115 (30–403)	124 (24–810)	0.001 *
**CALLY Index**	0.16 (0.01–17.3)	0.29 (0.01–21.1)	0.18 (0.01–21.3)	0.001 *
**HALP Score**	45.3 (3.91–218)	52.8 (11.2–189)	47.1 (3.91–218)	0.001 *
**PIV**	333 (20–4167)	286 (19–5021)	316 (19–5021)	0.013 *
**SIII**	564 (53–7063)	513 (55–5830)	554 (53–7063)	0.003 *
**SIRI**	1.24 (0.13–16.2)	1.04 (0.1–18.6)	1.16 (0.10–18.6)	0.007 *

DM, Diabetes mellitus; CRP, C-reactive protein; PLR, Platelet–lymphocyte ratio; PIV, Pan-immune inflammation value; SIII, Systemic immune-inflammation index; SIRI, Systemic Inflammation Response Index. * *p* < 0.05 is statically significant (bold).

**Table 4:** Comparison of parameters according to dysplasia.

	No-Dysplasia (ND), (n = 425)	Low-Grade Dysplasia (LGD), (n = 263)	High-Grade Dysplasia (HGD), (n = 70)	*p*
**Age (year)**	60.8 ± 12.4	60.7 ± 9.5	63.7 ± 9.5	0.182
**Glucose (mg/dL), (mean ± SD)**	109 (±48)	106 (±42)	102 (±34)	0.510
**Total protein (g/L)**	68 (36–89)	68 (41–81)	68 (43–76)	0.343
**Albumin (g/L)**	44 (19–53)	41 (24–49)	38 (26–47)	0.001 *
**CRP (mg/dL)**	4.6 (0.0–161)	5.4 (0.1–106)	6.5 (0.1–147)	0.116
**Neutrophil (10^9^/L)**	4.3 (0.6–28)	4.4 (1.6–21)	4.6 (1.6–23)	0.311
**Lymphocyte (10^9^/L)**	2.3 (0.4–6.6)	2.1 (0.1–6.8)	1.8 (0.3–5.9)	0.001 *
**Platelet (10^9^/L)**	272 (91–759)	265 (113–563)	276 (109–575)	0.670
**PLR**	121 (24–406)	123 (34–727)	165 (67–810)	0.001 *
**CALLY Index**	0.19 (0.0–21)	0.18 (0.0–20.8)	0.1 (0.01–7.3)	0.003 *
**HALP Score**	49.9 (5.9–218)	46.5 (3.9–211)	30.7 (5.9–144)	0.001 *
**PIV**	303 (19–4167)	316 (20–5021)	433 (46–3681)	0.017 *
**SIII**	543 (53–7063)	527 (113–4256)	750 (105–5815)	0.001 *
**SIRI**	1.09 (0.1–16)	1.2 (0.16–18)	1.48 (0.2–11.4)	0.001 *

* *p* < 0.05 is statically significant (bold). CRP, C-reactive protein; PLR, Platelet–lymphocyte ratio; PIV, Pan-immune inflammation value; SIII, Systemic immune-inflammation index; SIRI, Systemic Inflammation Response Index. Albumin difference: HGD-LGD = 0.01, HGD-ND < 0.000, LGD-HGD < 0.000. Lymphocyte difference: HGD-LGD = 0.04, HGD-ND = 0.001, LGD-HGD = 0.003. PLR difference: HGD-ND < 0.001, HGD-LGD = 0.01. CALLY index difference: HGD-ND = 0.005. HALP score difference: HGD-LGD < 0.001, HGD-ND < 0.001. PIV index difference: HGD-NO dysplasia = 0.014. SIII difference: HGD-ND < 0.001, HGD-LGD < 0.001. SIRI difference: HGD-ND < 0.001, HGD-LGD = 0.048.

**Table 5:** Indexes according to localization of colon polyps.

	Proximal Colon (n = 171)	Distal Colon (n = 501)	Proximal + Distal Colon (n = 86)	*p*
**PLR**	127 (24–372)	121 (30–810)	139 (59–700)	0.235
**CALLY index**	0.16 (0.01–21)	0.19 (0.01–20)	0.20 (0.01–14)	0.994
**HALP score**	46 (9.5–211)	47 (5.9–218)	47 (3.9–131)	0.571
**PIV**	328 (30–3239)	310 (19–4099)	361 (20–5021)	0.688
**SIII**	582 (53–3339)	531 (55–5830)	554 (139–7063)	0.764
**SIRI**	1.13 (1.15–7.7)	1.15 (0.1–11.4)	1.31 (0.13–18.6)	0.309

PLR, Platelet–lymphocyte ratio; PIV, Pan-immune inflammation value; SIII, Systemic immune-inflammation index; SIRI, Systemic Inflammation Response Index.

**Table 6:** Indexes according to the number of polyps.

	Solitary (n = 580)	Multiple (n = 178)	*p*
**PLR**	122 (24–727)	132 (35–810)	0.339
**CALLY index**	0.19 (0.01–21)	0.16 (0.01–20)	0.257
**HALP score**	47.6 (6.4–211)	45.5 (3.9–218)	0.275
**PIV**	314 (19–4099)	336 (20–5021)	0.539
**SIII**	552 (53–5830)	557 (92–7063)	0.555
**SIRI**	1.13 (0.1–10.4)	1.25 (0.13–18.6)	0.150

PLR, Platelet–lymphocyte ratio; PIV, Pan-immune inflammation value; SIII, Systemic immune-inflammation index; SIRI, Systemic Inflammation Response Index.

**Table 7:** Indexes according to the diameter of polyps.

	Polyp Size < 1 cm (n = 459)	Polyp Size ≥ 1 cm (n = 299)	*p*
**CALLY index**	0.18 (0.01–21)	0.17 (0.00–20)	0.256
**HALP score**	47.8 (8–218)	44.2 (3.9–211)	0.036 *
**PIV**	312 (19–4167)	333 (20–5021)	0.547
**SIII**	544 (55–7063)	569 (53–5830)	0.313
**SIRI**	1.14 (0.1–16)	1.22 (0.13–18)	0.294

PIV, Pan-immune inflammation value; SIII, Systemic immune-inflammation index; SIRI, Systemic Inflammation Response Index. * *p* < 0.05 is statically significant (bold).

**Table 8:** Logistic regression analysis of risk factors for the development of neoplastic polyps.

Step 1	B	*p*	OR	95% CI for EXP(B)	
				Lower	Upper
**Polyp diameter (≥1 cm)**	1.001	0.000	2.721	1.624	4.559
**CALLY index**	−0.076	0.008	0.927	0.876	0.980
**HALP score**	−0.008	0.035	0.992	0.986	0.999
**PIV**	−0.001	0.269	0.999	0.998	1.000
**SIII**	0.000	0.413	1.000	1.000	1.001
**SIRI**	0.025	0.870	1.025	0.764	1.375

PIV, Pan-immune inflammation value; SIII, Systemic immune-inflammation index; SIRI, Systemic Inflammation Response Index.

**Table 9:** Positive and negative predictive values (PPV, NPV) of new indexes.

	PPV (%)	NPV (%)
**PLR**	71.5	36.3
**CALLY Index**	79.4	33.0
**HALP Score**	71.7	36.5
**PIV**	70.8	35.4
**SIII**	71.6	36.2
**SIRI**	72.6	37.3

PLR, Platelet–lymphocyte ratio; PIV, Pan-immune inflammation value; SIII, Systemic immune-inflammation index; SIRI, Systemic Inflammation Response Index.

## Data Availability

All data generated or analyzed during this study are included in this article. The datasets in the current study are available from the corresponding author upon reasonable request.
